# Grand Challenges in Bioinformatics Data Visualization

**DOI:** 10.3389/fbinf.2021.669186

**Published:** 2021-06-17

**Authors:** Seán I. O'Donoghue

**Affiliations:** ^1^ Garvan Institute of Medical Research, Darlinghurst, NSW, Australia; ^2^ School of Biotechnology and Biomolecular Sciences, University of New South Wales, Kensington, NSW, Australia; ^3^ CSIRO Data61, Eveleigh, NSW, Australia

**Keywords:** bioinformatics, data science, data visualization, visual analytics, computational biology, science communication

## Introduction

Increasingly, the life sciences rely on data science, an emerging discipline in which visualization plays a critical role. Visualization is particularly important with challenging data from cutting-edge experimental techniques, such as 3D genomics, spatial transcriptomics, 3D proteomics, epiproteomics, high-throughput imaging, and metagenomics. Data visualization also plays an increasing role in how research is communicated. Some scientists still think of data visualization as optional; however, as more realize it is an essential tool for revealing insights buried in complex data, bioinformatics visualization is emerging as a subdiscipline. This article outlines current and future grand challenges in bioinformatics data visualization, and announces the first publication venue dedicated to this subdiscipline.

Over the past two decades, life science data have increased rapidly in volume and complexity, with the result that data analysis is often the major bottleneck (O’Donoghue et al., 2010a). For example, “*All major genomics breakthroughs so far have been accompanied by the development of ground-breaking statistical and computational methods*” (Green et al., 2020). Thus, in the remaining decades of the 21st century, life scientists will become increasingly reliant on the emerging tools and methods of data science (Blei and Smyth, 2017; Altman and Levitt, 2018).

**FIGURE 1:**
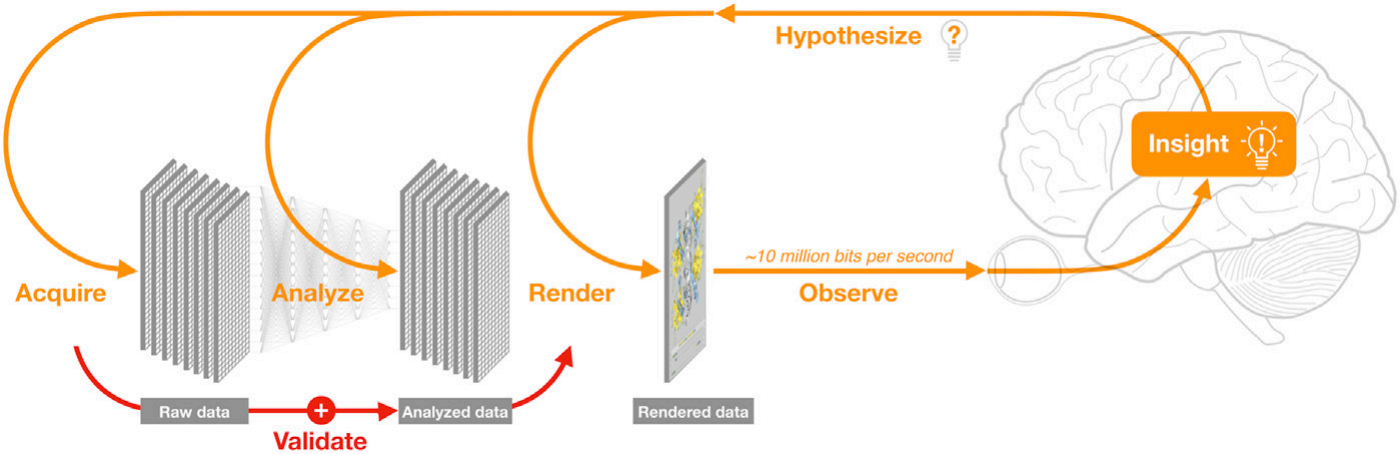
The data science cycle. Analysis of newly acquired data increasingly relies on integration with large, accumulating volumes of complex, pre-existing data, and requires frequent re-analysis and re-rendering. Visualization is the main way researchers observe both raw and analysed data; an overarching grand challenge of data visualization is to leverage human visual capabilities—which involve most of the brain and can process ∼10 million bits per second (Koch et al., 2006) to recognize patterns within ∼100 ms (Healey and Enns, 2012) — thereby transforming data into insight. These insights, in turn, lead to new hypotheses, thus continuing the cycle. Unfortunately, the critical step of manually validating derived models by visually comparing raw vs. analysed data (Anscombe, 1973) is often overlooked.

One of these methods is data visualization (a.k.a. DataVis), which plays a critical role in transforming data and analysis outcomes into insight (Card et al., 1999). Data visualization involves analysis, design, and rendering, as well as observation and cognitive processing (Figure 1). Some scientists think of DataVis as an optional step mostly aimed at aesthetics — however, there is growing recognition that it is an essential tool in the analysis of complex data; two indicators of this recognition are the recent sales of DataVis companies Looker and Tableau for US$3B and $16B, respectively.

Currently, however, most attention is focused on another aspect of data science, namely, the use of machine learning to develop artificial intelligence systems. Such systems have recently led to exciting advances in the life sciences (e.g., Callaway, 2020a) — but also to some hyperbole. Clearly, machine learning methods are increasingly critical for research; but these methods also have limitations (Challen et al., 2019; Heaven, 2019; Yu and Kohane, 2019). More fundamentally, automated methods are insufficient, since analysis outcomes must be observed and understood by an analyst before insight can occur (Figure 1). Most analysts use data visualization as an integral part of their cognitive processes—especially important is manual validation, which involves checking for errors and outliers in raw data, and for wrong assumptions used in automated analysis methods (Anscombe, 1973).

Automated data analysis (including machine learning) and data visualization are just components of the larger goal of data science, which the eminent computer scientist Fred Brooks argues should focus on ‘Intelligence Amplification’ (a.k.a. I.A.) — i.e., on amplifying our abilities to manage more complex data (Brooks, 1996). In my opinion, helping achieve the goal of I.A. is the overarching grand challenge of DataVis.

## Prioritizing Grand Challenges in Bioinformatics

Since data visualization aims to amplify human intelligence, we could ask ourselves^1^: of all our colleagues working across different life sciences, whose intelligence most needs amplifying? Or, humor aside, which fields are creating data that are both important and urgently need improvements in visual analysis?

**FIGURE 2:**
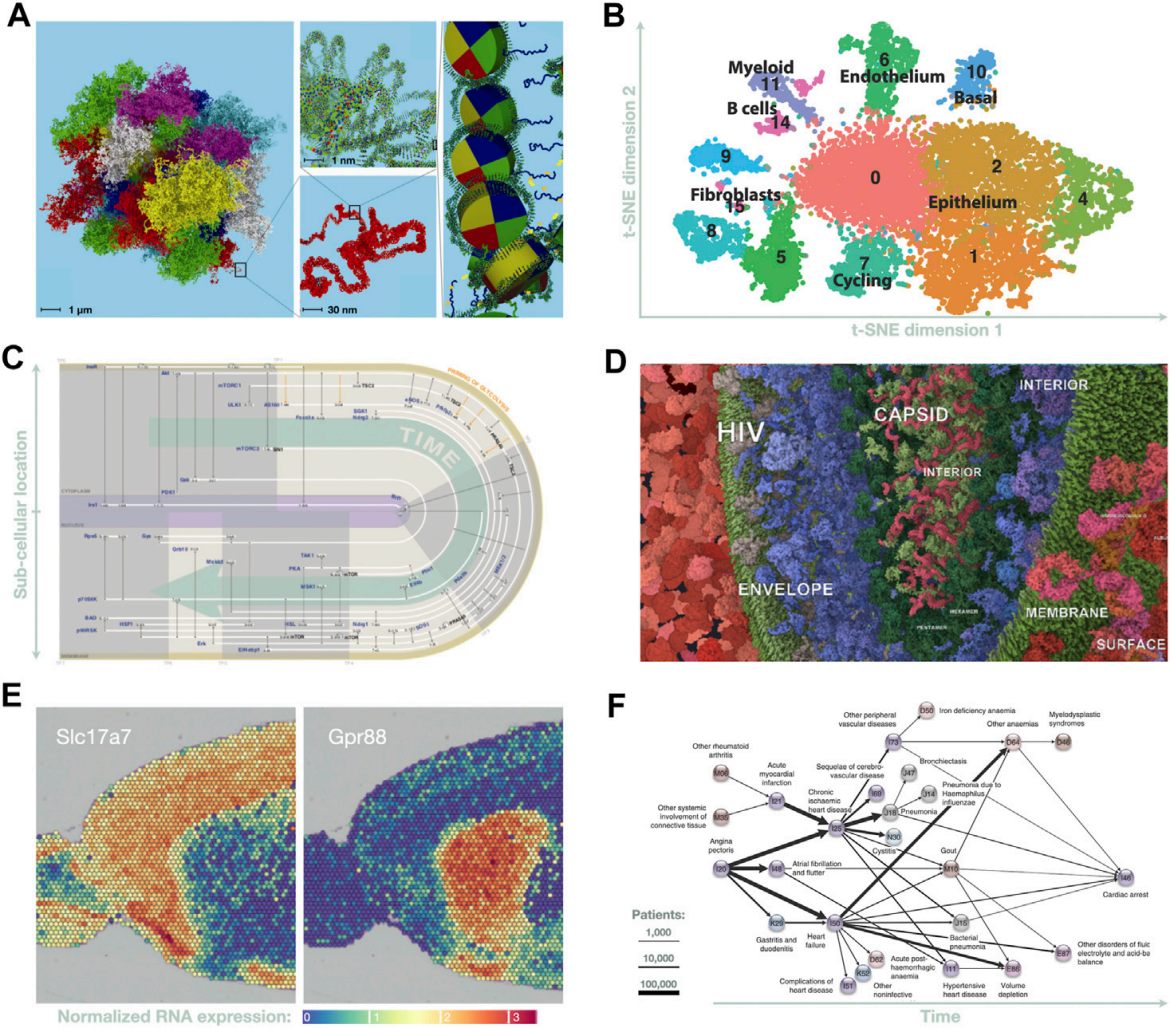
Six data visualization methods currently used in bioinformatics grand challenges. **(A)** A molecular-scale, 3D model of the human genome may soon be feasible; in preparation, visualization tools are being developed to enable interactive, multiscale exploration of such models (adapted from Asbury et al. (2010)). **(B)** t-SNE analysis of scRNA-seq data on breast cancer metastasis (adapted from Valdes-Mora et al. (2019)). **(C)** Spatiotemporal graph of phosphorylation events in fat cells following insulin stimulation (adapted from Ma et al. (2015), by Jenny Vuong). **(D)** Here, an interactive, web-based data integration environment is used to explore and curate a molecular-scale model of a subcellular landscape showing HIV-host interactions (Klein et al., 2018; Autin et al., 2020). **(E)** Portion of spatial transcriptomics analysis showing expression of two genes in an anterior slice from mouse brain (adapted from Vandenbon and Diez (2020)). **(F)** Disease trajectory graph showing progression from heart pain to cardiac arrest across the entire Danish population (adapted from Jensen et al. (2014)).

Addressing this question is the core mission of an annual series of international meetings on ‘Visualizing Biological Data’ (VIZBI^2^). From my perspective as chair of this meeting series, it is clear that the biological and biomedical sciences are currently awash with vexing data challenges where current analysis methods and tools are fundamentally inadequate. Thus, researchers looking for grand challenges in bioinformatics data visualization (a.k.a. BioVis) are spoilt for choice; of very many worthy challenges, below are six that have been highlighted repeatedly by VIZBI speakers over the past decade, as cases in which innovations in visual analysis are likely to lead to significant breakthroughs in our understanding of life. Figure 2 showcases some of the visual methods currently being used to partly address these challenges. This list of challenges is far from comprehensive; researchers already focused in a particular field of the life sciences (e.g., drug design, medicine, ecology) would likely prioritize very different lists of worthy challenges.

I. In genomics, there is rapid progress towards the goal of determining the spatiotemporal organization of chromosomes at molecular-scale resolution (Figure 2A); this is driven by advances in sequencing technologies that can infer spatial contacts (Lieberman-Aiden et al., 2009), as well as in high resolution imaging (Ou et al., 2017). Integrating these multiscale and multimodal data poses formidable visualization challenges (Ay and Noble, 2015; Serra et al., 2015); however, achieving this goal would transform our understanding of what gets transcribed, and how and when transcription is controlled in different cell types.

II. In transcriptomics, rapid advances in single-cell RNA-seq (scRNA-seq) techniques now make it possible to track behaviors of individual cells in unprecedented detail, providing a window into events that were previously hidden (Wills et al., 2013). For example, scRNA-seq can be used to track cell differentiation and the evolution of cell-cell contacts during the growth of cancerous tumours (e.g., Valdes-Mora et al., 2019). Also, in combination with imaging methods, these techniques can be used to resolve the spatial location of RNA transcripts within single cells (Chen et al., 2015). Each type of scRNA-seq experiment produces data that are both highly multidimensional but also very specific; tailoring effective data analysis strategies for each type of experiment requires development of innovative visual analysis methods to overcome limitations with existing, generic approaches such as t-SNE (‘t-distributed stochastic neighbor embedding’; Figure 2B) (Van der Maaten and Hinton, 2008) or diffusion maps (Coifman et al., 2005). This challenge currently engages many bioinformaticians, driven by the promise of discovering the key mechanisms used to control cellular processes.

III. In proteomics, advances in high-throughput mass-spectroscopy (Kim et al., 2006; Morelle et al., 2006; Olsen et al., 2006) have begun to provide first glimpses into the highly dynamic epiproteome, i.e., the set of all post-translational modifications (PTMs) made to all proteins in a cell (Zheng et al., 2016; Kaur et al., 2019). So far, at least 200 distinct types of PTMs are known^3^, and related advances are revealing that comparable levels of complexity occur in modifications seen to both RNA (Roundtree et al., 2017) and lipids (Shevchenko and Simons, 2010). Currently, most of these modifications are poorly studied; even phosphorylation of human proteins—one of the best studied PTMs—gives rise to a phosphoproteome that is still largely unknown or ‘dark’ (Needham et al., 2019). However, this is set to change rapidly over the next few years, although extracting insights from the dynamic, highly multidimensional datasets from epiproteomics (Figure 2C), epitranscriptomics, and lipidomics remains a major challenge (Kaur et al., 2019; Kaur et al., 2020). Nonetheless, the insights gained are likely to fundamentally advance our understanding of cellular processes in health and diseases—for example, by revealing molecular events that occur during illness or following therapeutic interventions.

IV. In cell biology, a convergence of several experimental techniques and computational methods are driving work towards an audacious goal: determining the spatiotemporal organization of a human cell at molecular resolution (Tomita, 2001; Singla et al., 2018). The spatial location of proteins can be mapped at sub-cellular resolution using imaging and mass-spectrometry techniques (Boisvert et al., 2012; Gatto et al., 2019; Lundberg and Borner, 2019); the molecular structure of these proteins can be determined using cryogenic electron microscopy (Bai et al., 2015; Callaway, 2020b) — even when they occur in large complexes. Transient protein complexes can be either measured experimentally, inferred from sequence information (Elofsson, 2021), or modelled in large-scale molecular simulations (e.g., McGuffee and Elcock, 2010; Feig et al., 2015). Still largely unmet (Figure 2D) is the formidable challenge of developing visual methods that integrate these data with information on protein-protein interactions (Gehlenborg et al., 2010; Ghosh et al., 2011), protein-small molecule interactions (Krone et al., 2016), protein 3D structure (O’Donoghue et al., 2010b; Johnson et al., 2015; Kozlíková et al., 2017; Olson, 2018), and protein dynamics (Humphrey et al., 1996; Rysavy et al., 2014; Ferina and Daggett, 2019). If this challenge can be met, this would provide a structural framework for understanding the molecular basis of cell behavior; this, in turn, could have profound impact, similar to how the structure of DNA advanced our understanding of the molecular basis of information storage and replication (Watson and Crick, 1953).

V. Multiple advances in tissue-scale imaging are driving other audacious goals: for example, two-photon fluorescence microscopy (Pittet and Weissleder, 2011) is being used to construct 3D maps of neural connectivity in mammalian brains (e.g., Economo et al., 2016), and also to track real-time movements of cells and subcellular structures within living tissues, including tumors (e.g., Kedrin et al., 2008; Conway et al., 2018). In addition, combining tissue imaging with fluorescence *in situ* hybridization methods now enables spatial mapping of RNA transcription (Ståhl et al., 2016; Burgess, 2019) at near-cellular resolution (Figure 2E) (Stickels et al., 2020; Marx, 2021). Combining these data with tissue-scale or whole-body kinetic modeling (Alqahtani, 2017) has potential to revolutionize our understanding of physiology and the body’s responses to events such as tumor growth or therapeutic interventions. However, extracting insight from such massive, complex datasets requires development of highly tailored, innovative visual analysis methods (e.g., Santos et al., 2015; GTEx Consortium, 2017; Uhlen et al., 2017) to address the many challenges of bridging molecular information with tissue- and whole-organism scale data (Walter et al., 2010; O’Donoghue et al., 2018).

VI. Finally, a set of daunting challenges lie in comparing temporal changes in clinical records across cohorts (Karczewski and Snyder, 2018). The complexity and volume of these data are increasing rapidly due to wearable devices (Kim et al., 2019; Ray et al., 2019); however, data dimensionality dramatically increases when microbiome analysis is also included (e.g., Schüssler-Fiorenza Rose et al., 2019). Current visual analysis methods are often inadequate even when exploring the microbiome of a single person (Procter et al., 2010; Pasolli et al., 2019). Addressing these many challenges calls for innovative new approaches in how we visualize phylogenetic (e.g., Rosindell and Harmon, 2012; Letunic and Bork, 2019) and pan-genomic relationships (e.g., Ding et al., 2018), how we compare microbiomes (e.g., Caporaso et al., 2010; Darling, 2004; Waterhouse et al., 2009), and how we explore clinical information gathered from large cohorts (Figure 2F) (e.g., Jensen et al., 2014).

## Communicating Science Visually

Once any of the above grand challenges are addressed, a new challenge is created: how to convey the significance of this breakthrough to others. *“Science isn’t complete until it’s communicated”* (Day, 1998); but the highly specific nature of the life sciences can make it difficult to communicate a breakthrough even to researchers working in closely related fields, let alone to the general public. Here again data visualization plays an increasingly central role. Many of the visualization methods and tools designed for analysis can be repurposed for communication; but often dedicated communication approaches need to be developed to address specific data challenges, especially when conveying complex or unfamiliar ideas.

For example, an intrinsic difficulty with communicating insights from a molecular-scale model of a human cell (challenge IV, above) is that ‘mesoscale’ molecules (Johnson et al., 2015; Goodsell et al., 2018) behave very differently to macroscopic objects. This difficulty is driving development of innovative communication approaches to convey these dynamic behaviors, e.g., via 2D illustration (e.g., Gardner et al., 2018) or 3D graphics (Goodsell et al., 2020; e.g., Muzic et al., 2015; Waldin et al., 2019). In turn, such methods are being used to create informative and inspiring videos^4^ (McGill, 2008; Johnson and Hertig, 2014; Iwasa, 2015) and to build interactive environments that can be explored with virtual reality techniques (Johnston et al., 2018).

In contrast to visual analysis, subjective qualities such as aesthetics and novelty become important when using visual methods for outreach. However, the impact of visual storytelling goes beyond outreach; the difficult process of assembling our hypotheses into clear, visual narratives (Nayak et al., 2020) invariably involves integrating pre-existing data in new ways, often revealing hidden assumptions and knowledge gaps. This, in turn, often leads to new insights and hypotheses (e.g., Reilly and Ingber, 2017), thereby continuing the data science cycle (Figure 1). Thus, visual communication should also be considered as an intrinsic part of any grand challenge in bioinformatics data visualization.

## Bridging Bioinformatics and Visualization Research

Addressing the above grand challenges requires combining expertise in visual analysis with specific knowledge about the biological context of each experiment, and about what can be inferred, given expected errors and given prior knowledge. This, in turn, requires an exchange of knowledge between researchers in computer science and in various life sciences. Unfortunately, these communities rarely attend the same meetings, have very different publication practices, and are strongly disincentivized to collaborate, since their work performance and funding are assessed using fundamentally different metrics.

To help counteract these obstacles, a range of resources have been created for life scientists that showcase how data visualization is transforming biology; these include: special issues of Nature Methods^5^ (Evanko, 2010) and of the Journal of Molecular Biology^6^; a section of BMC Bioinformatics dedicated to advances in either data visualization or image analysis^7^; a Nature Methods article series on visualization issues^8^; and the VIZBI^9^ meeting series (mentioned above), which was highlighted in Nature News (Callaway, 2016). Corresponding resources have also been created for computer scientists, including several Dagstuhl reports (Görg et al., 2013; Aerts et al., 2018) and regular meetings, including the VCBM^10^ (‘Visual Computing for Biology and Medicine’) and BioVis^11^ workshops, co-located with the annual conferences Eurographics^12^ and ISMB^13^/IEEE VIS^14^, respectively. In addition, other international meetings bridge related communities, but with more targeted focus—for example, biomedical data visualization (Holzinger, 2012; O’Donoghue et al., 2018) is the focus of MediVis^15^, while molecular graphics (Olson, 2018; Martinez et al., 2019) is the focus of MolVA^16^ and of several Shonan meetings (Schafferhans et al., 2016; Baaden et al., 2018).

These activities have catalyzed adoption of emerging visualization methods in the life sciences; notable examples include: use of ggplot2 (Wickham, 2009) for offline analysis, use of D3 (Bostock et al., 2011) for interactive data exploration in websites; use of virtual reality in molecular graphics (Muzic et al., 2015; Sommer et al., 2018); use of augmented reality in surgery (Bernhardt et al., 2017; Maier-Hein et al., 2017); and the use of interactive volume rendering for full-body virtual autopsies (Ljung et al., 2006).

Looking forward, advances in computer hardware and software are set to provide greatly improved graphics as well as new paradigms for user interaction. One of the overarching grand challenges in BioVis is to use these advances to improve research, communication, training, and clinical practices.

## Publishing Advances in Bioinformatics Data Visualization

As with all frontier, interdisciplinary work, publishing advances in BioVis can be problematic. Publication venues in computer science often reject manuscripts describing novel methods that lack broad applicability, or describing novel tools that lack a user study—even when the advances described are obviously of very high value to domain experts. Life science journals often reject manuscripts describing novel visual analysis approaches that are too technical or have not yet been used to derive significant, novel biological insights—even when the advances described are highly innovative or required enormous effort. Even journals specializing in bioinformatics often reject manuscripts that describe user studies, design studies, or improvements to existing tools.

Advances in BioVis could lead to tremendous impact, by improving the tools used by life science researchers. However, publication decisions are often driven by perceived potential impact, a criterion that frequently rejects even the most ground-breaking work (Bjørk, 2020). To address this issue, several open-access publishers such as Frontiers^17^, BMC^18^, and PeerJ^19^ have emerged in the past decades with the mandate to base publication decisions solely on scientific rigor and reproducibility.

As part of this process, this ‘grand challenge’ article has been written to accompany the launch of the Data Visualization section in the newly created journal Frontiers in Bioinformatics—the first publication venue dedicated to bioinformatics data visualization. By collecting advances across all life sciences, the Data Visualization section will facilitate exchange of knowledge and best practises between research groups that may otherwise never cross paths; mostly, these groups will comprise bioinformaticians, biomedical researchers, computer scientists, and science communicators—but BioVis also engages educators, user-experience designers, as well as visual arts practitioners, particularly graphic designers and medical illustrators.

## Conclusion

It is fortunate that bioinformatics data visualization engages a broad community with diverse backgrounds and perspectives, since one of our core processes is to overcome current cognitive biases in analysis, and to find more effective ways of seeing, analyzing, and thinking about our data. A historical exemplar of this process is the inspiring, interdisciplinary work of Jane and David Richardson^20^, who devised a method for transforming complex, all-atom representations of large protein structural models into ribbon representations that are greatly simplified and often insightful (Richardson, 1981; Richardson and Richardson, 1989).

Our task going forward is to find analogous ways to reimagine the much larger and more complex datasets in today’s grand challenges, and—using current and future advances in computer graphics—to invent simplifying transformations that are also insightful. Each such invention can be thought of as a step in creating a new visual language (Lima, 2011) that will enhance how we explore, describe, and communicate the processes of life.

Given the daunting data challenges already at hand, creating this visual language will likely be difficult, and will require considerable creativity combined with statistical and mathematical rigor. Given the even more daunting data challenges that are set to come, we could say that bioinformatics data visualization has barely begun (O’Donoghue et al., 2010a).

## Footnotes

1 Paraphrasing from Fred Brooks (1996).

2
https://vizbi.org/

3
https://www.uniprot.org/docs/ptmlist

4
https://clarafi.com/showcase/

5
https://www.nature.com/collections/iecaaechei

6
https://www.sciencedirect.com/journal/journal-of-molecular-biology/special-issue/10VZQRR6PS7

7
https://bmcbioinformatics.biomedcentral.com/articles/sections/imaging-image-analysis-and-data-visualization

8
http://blogs.nature.com/methagora/2013/07/data-visualization-points-of-view.html

9
https://vizbi.org/

10
https://dblp.org/db/conf/vcbm

11
http://biovis.net/

12
https://www.eg.org/

13
https://www.iscb.org/about-ismb

14
http://ieeevis.org/

15
http://www.graphicslink.co.uk/MediVis2021/

16
http://decibel.fi.muni.cz/∼xbyska/molva/

17 “Frontiers does not reject manuscripts based on their perceived potential impact” - https://www.frontiersin.org/about/review-system

18 “We do not make editorial decisions on the basis of the interest of a study or its likely impact” - https://bmcbioinformatics.biomedcentral.com/about

19
https://peerj.com/about/FAQ/#quality

20
https://stories.duke.edu/sciences-mother-of-ribbon-diagrams-celebrates-50-years-at-duke
